# Autotaxin and Lipoprotein Metabolism in Calcific Aortic Valve Disease

**DOI:** 10.3389/fcvm.2019.00018

**Published:** 2019-03-01

**Authors:** Patrick Mathieu, Marie-Chloé Boulanger

**Affiliations:** Laboratory of Cardiovascular Pathobiology, Department of Surgery, Research Center, Quebec Heart and Lung Institute, Laval University, Quebec, QC, Canada

**Keywords:** aortic valve, calcific aortic stenosis, autotaxin (ATX), inflammation, phospholipid (PL)

## Abstract

Calcific aortic valve disease (CAVD) is a complex trait disorder characterized by calcific remodeling of leaflets. Genome-wide association (GWA) study and Mendelian randomization (MR) have highlighted that *LPA*, which encodes for apolipoprotein(a) [apo(a)], is causally associated with CAVD. Apo(a) is the protein component of Lp(a), a LDL-like particle, which transports oxidized phospholipids (OxPLs). Autotaxin (ATX), which is encoded by *ENPP2*, is a member of the ecto-nucleotidase family of enzymes, which is, however, a lysophospholipase. As such, ATX converts phospholipids into lysophosphatidic acid (LysoPA), a metabolite with potent and diverse biological properties. Studies have recently underlined that ATX is enriched in the Lp(a) lipid fraction. Functional experiments and data obtained in mouse models suggest that ATX mediates inflammation and mineralization of the aortic valve. Recent findings also indicate that epigenetically-driven processes lower the expression of phospholipid phosphatase 3 (*PLPP3*) and increased LysoPA signaling and inflammation in the aortic valve during CAVD. These recent data thus provide novel insights about how lipoproteins mediate the development of CAVD. Herein, we review the implication of lipoproteins in CAVD and examine the role of ATX in promoting the osteogenic transition of valve interstitial cells (VICs).

## Introduction

Calcific aortic valve disease (CAVD) is a prevalent cardiovascular disorder. Fibro-calcific remodeling is one key process involved in the development of CAVD. During CAVD, fibrosis and mineralization of the aortic valve (AV) cusps progressively lead to a narrowing of the aortic orifice, a clinical condition referred to as aortic valve stenosis (1). Different risk factors such as age, male gender, hypertension, diabetes, and bicuspid aortic valve (BAV) have been associated with CAVD (2). Studies have highlighted that osteogenic transition of valve interstitial cells (VICs) is a predominant feature in surgically explanted aortic valves (3, 4). Inflammation and growth factors are active players that promote the synthesis of extracellular matrix (ECM) and also trigger an osteogenic program (5). For instance, mice deficient for interleukin 1 receptor antagonist (*Il1rn*), a natural circulating inhibitor of interleukin 1 beta (Il1b), develop a thickening of the aortic valve and a rise of transaortic velocities (6). VIC, a heterogeneous mesenchymal cell population, has a high degree of plasticity in response to environmental cues (7). As such, different environmental signals along with genetic predisposition are likely interacting in driving the osteogenic response of VICs.

Familial aggregation observed in CAVD has underlined that a strong genetic component is involved in the development of this valve disorder (8). Though the genetic architecture of CAVD is just emerging, genome-wide association (GWA) studies and Mendelian randomization (MR) analyses have underlined in the last 5 years that *LPA*, which encodes for apoliprotein(a) [apo(a)], is causally associated with CAVD (9, 10). This finding has fuelled considerable interest to decipher the molecular processes whereby lipoprotein(a) [Lp(a)] promotes the development of CAVD. Recent findings have shed some light on this issue and suggest that an enzymatic pathway along with Lp(a) promote inflammation and osteogenesis in the aortic valve (11). Isolated Lp(a) fraction showed an enrichment for autotaxin (ATX), a lysophospholipase D, which is transported in the aortic valve (12). ATX is a ubiquitous enzyme that transforms oxidized phospholipids (OxPLs), a significant cargo transported by Lp(a), into lysophosphatidic acid (LysoPA). LysoPA is a highly active metabolite and promotes inflammation, fibrosis and osteogenesis (13). Herein, we are reviewing the genetic association of *LPA* with CAVD and the underpinning processes whereby this lipoprotein promotes the development of CAVD through ATX-LysoPA pathway.

## Genetics, *LPA* and CAVD

Previous work conducted in the western part of France underscored that CAVD is clustering in some extended families (14). More recently, nationwide epidemiological assessment in Sweden reported an increased risk of developing CAVD among subjects with at least one sibling diagnosed with the disorder (15). The risk was magnified among siblings (HR = 3.4) as compared to spouses of index cases (HR = 1.2), suggesting that the genetic component is likely having a stronger effect compared to shared environmental risk factors (15). Rare mutations in *NOTCH1* have been documented in some families with BAV, an abnormal valve configuration with two cusps instead of three and a risk factor for CAVD (16). However, rare mutations in *NOTCH1* only explain a small fraction of cases. In the last decade, GWA studies, which test genetic associations between common single nucleotide polymorphisms (SNPs) and traits/disorders, have underlined the genetic architecture of these traits and have fuelled the development of novel therapies. Though the genetic architecture of CAVD is just emerging, recent findings have transformed the field and have helped to pinpoint causal pathways (17). Thanassoulis et al. reported in the first GWA study conducted on CAVD that a common gene variant rs10455872 (MAF = 0.07 in European population), which is located in *LPA*, reached genome-wide significance (9). The *LPA* locus is complex and includes copy number variants (CNVs) in the region encoding for kringle IV type 2 domain (KIV_2_), which is inversely related to the circulating level of Lp(a) (18). The index SNP at the *LPA* locus rs10455872 is associated with the number of KIV_2_ repeats and with plasma level of Lp(a). It is worth highlighting that plasma level of Lp(a) is largely determined by genetic factors and heritability may explain up to 90% of the variance (19). In a MR study design, *LPA* was identified as a plausible causal candidate in CAVD (9). These findings were corroborated in different studies (10, 20). However, a recent GWA study found that rs10455872 was not associated with congenital BAV, a common cause of CAVD (21). In a large meta-analysis including 1,797 CAVD cases and 131,932 controls, carriers of rs10455872 had a 1.66-fold risk of developing CAVD (22). Also, genetically-determined lower level of Lp(a) has been shown to reduce the risk of CAVD by 37% (23). Among subjects of European ancestry, the population attributable risk for Lp(a) in CAVD is 13% (24). These findings thus indicate that decreasing Lp(a) and/or blocking specific pathway(s) whereby this lipoprotein promotes CAVD could possibly translate into therapies in at-risk individuals.

## Oxidized Phospholipids and Lp(a)

Apo(a) is a highly polymorphic lipoprotein, which is linked to apolipoprotein B(apoB) moiety of low-density lipoprotein (LDL) by a disulfide bridge (25). Lp(a) is thus a LDL-like particle with an additional lipoprotein. However, some distinctive features characterize Lp(a). Among those, the cargo of Lp(a) includes a significant proportion of OxPLs, which binds to apo(a) moiety (26). Specifically, KIV_10_ domain of apo(a) is attached covalently to OxPL (26). Of note, genetically-determined level of OxPL linked to apo(a) [OxPL-apo(a)] increases the risk of CAVD by 1.09-fold (27). Also, the circulating levels of Lp(a) and OxPL are associated with a faster progression of aortic valve stenosis (28). OxPL is considered as a danger associated molecular pattern (DAMP), which is recognized by the innate immune system and is a potent trigger for the inflammatory process(29, 30).

## Development of CAVD: Osteogenic Transition and Inflammation

One key feature in CAVD is the transition of VICs toward osteoblast-like cells. One of the first step involves the reprograming of cells into activated VICs, which express myofibroblast markers such as alpha smooth muscle actin (*ACTA2*) and secrete ECM components such as collagen and remodeling enzymes (31). Transforming growth factor beta 1 (*TGFB1*) is expressed during the early stages of CAVD and promotes the transformation of quiescent cells into activated VICs (32). Secretion of bone morphogenetic protein 2 (BMP2) is a powerful stimulus that instigate an osteogenic program and the deposition of mineralized matrix (33). Key signaling pathways such as NOTCH1 and WNT/beta catenin are involved and promote the expression of osteogenic genes in VICs (34). NOTCH1 is a crucial regulator of osteogenic fate and lower signaling through this cascade increases the expression of *BMP2* (35). The osteogenic reprograming is tightly associated with the expression of key transcription factors (TFs) such as *RUNX2*, which coordinates the expression of several potent promoters of mineralization such as tissue non-specific alkaline phosphatase (*ALPL*). *ALPL* along with other ecto-nucleotidases, such as *ENPP1*, are overexpressed in surgically explanted mineralized aortic valves (36). ALPL and ecto-nucleotidases are embedded in matrix vesicles, which are secreted by VICs and promote the mineralization of the ECM by controlling the production of inorganic phosphate (37). Collagen type 1, secreted by VICs, contributes to the stiffening of the ECM and also serve as scaffold for biomineralization (38, 39).

Different pathobiological processes that involve ectopic biomineralization are associated with inflammation. It is likely that in response to diverse pathogenic stimuli some tissues respond by secreting mineralized matrix as a defensive mechanism. Hence, the production of biomineralized matrix can be interpreted as a response to injury (40). Hence, DAMPs and pathogen-associated molecular pattern (PAMP) receptors, which are expressed by VICs, contribute to activate cells and to promote a fibro-calcific response (41). Activation of toll-like receptors 2 and 4 (TLR2/4) promote a potent inflammatory response and the mineralization of isolated VIC cultures (42). Moreover, increased oxidative stress related to the uncoupling of nitric oxide synthase (NOS) and the overactivation of NADPH oxidase (NOX) during CAVD contribute to exacerbate the inflammatory response (43, 44). VICs secrete interleukin-6 (IL6), which potently induces ECM production and the expression of *BMP2* (45). IL6 also promotes the endothelial-to-mesenchymal transition (endoMT) of aortic valve endothelial cells into activated VICs (46). Histological examination of explanted mineralized AVs has consistently revealed the presence of macrophages and some T cells (47). In surgically explanted mineralized aortic valves, clonally expanded population of T cells is present and suggests that immunity is associated with the development of CAVD (48). In human explanted AVs, the number of inflammatory cell clusters is associated with the degree of tissue remodeling (47). These data, thus, highlight that inflammation and osteogenesis are intertwined together during the development of CAVD. However, it is worth pointing out that despite mounting evidence for a participation of inflammation to CAVD, it is presently unclear if it plays a causal role in the development and progression of this disorder. Further work is needed to identify key causal drivers in CAVD.

## Autotaxin

**Figure 1:**
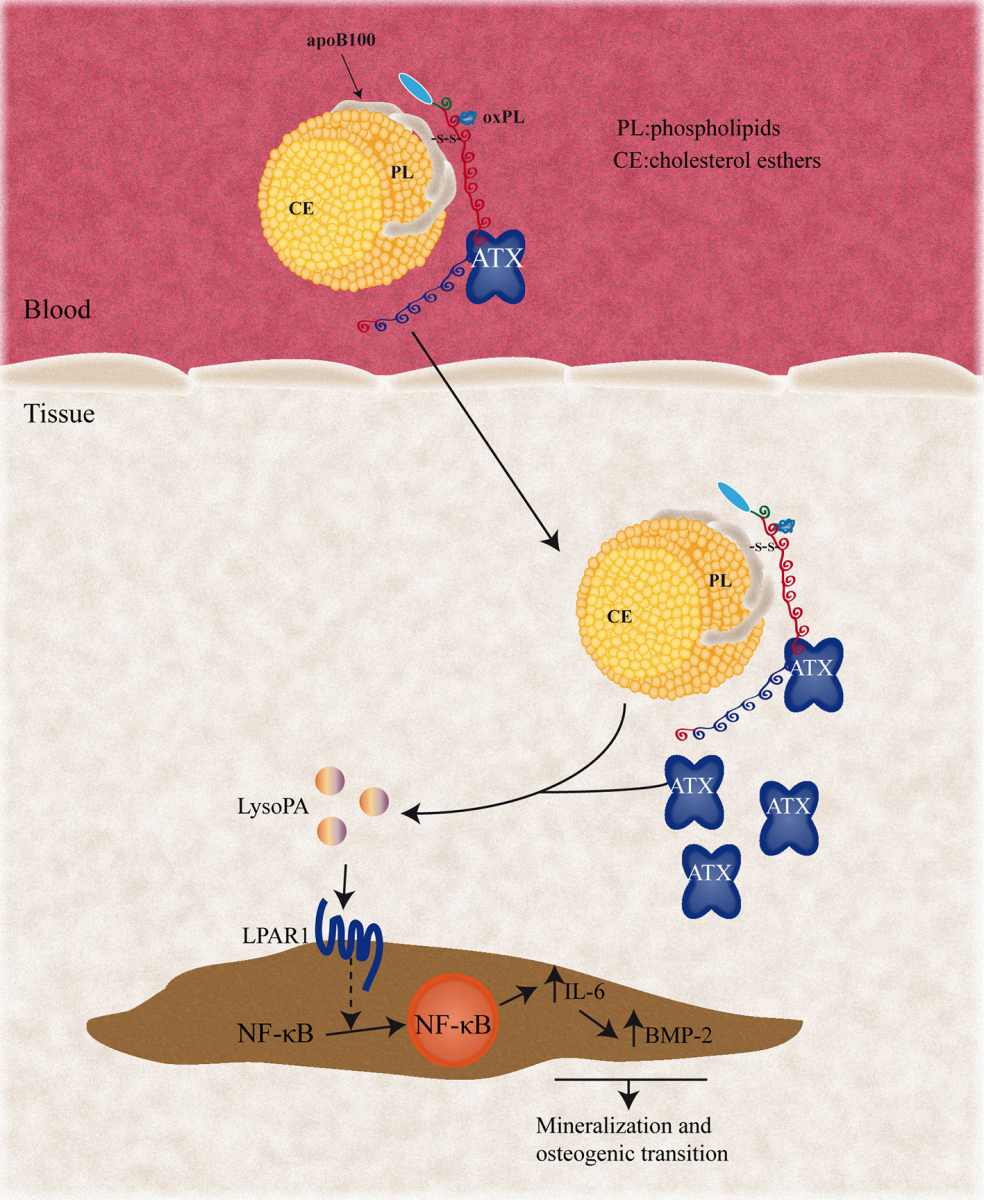
Circulating Lp(a) particles associate with ATX and enter the aortic valve, where ATX can metabolize lysophosphatidylcholine (LysoPC) presents in the Lp(a) particles into lysophosphatidic acid (LysoPA). Pericellular LysoPA can then associate with LPAR1, which promotes NF-κB nuclear translocation. NF-κB activation results in elevated expression of the IL-6 and BMP-2 pro-osteogenic factors. ATX, autotaxin; LPAR1, lysophosphatidic acid receptor 1; IL-6, interleukin 6; BMP2, bone morphogenetic protein 2; NF-κB, nuclear factor kappa B.

ATX, which is encoded by *ENPP2*, is a lysophospholipase D enzyme that was initially identified in melanoma cell line as a motility factor (49). It transforms lysophosphatidylcholine (LysoPC) into LysoPA. Oxidative transformation of lipoproteins generates significant amount of LysoPC through a lipoprotein-associated phospholipase A2 pathway (50). Mahmut et al. showed that LysoPC is a reactive metabolite that promoted an osteogenic program in VICs (51). However, subsequent analysis by using thin layer chromatography showed in explanted mineralized AVs that the amount of LysoPA surpassed by manifold the level of LysoPC (12). Chromatography-tandem mass spectrometry performed in explanted mineralized AVs confirmed the presence of LysoPA with a predominance of 16:0 and 18:1 species (52). These data, thus, suggested that active processes were likely at play in promoting the production of LysoPA in the AV (Figure 1). Measurements performed in control non-mineralized and explanted CAVD tissues showed that enzymatic activity of ATX was increased by 60% in mineralized cusps (12). In the latter study, ATX activity in mineralized aortic valves was increased in both tricuspid and BAVs. Confocal analysis of mineralized aortic valves also demonstrated that immunofluorescence for apo(a) was co-distributed with ATX (12). ATX activity was enriched in isolated Lp(a) fraction and proximity ligation assay in aortic valves confirmed the interaction between ATX and apo(a). Moreover, we found a high level of ATX in VICs, which was secreted in response to LysoPC and OxPLs treatment. Further experiments showed that LysoPC-induced VIC culture mineralization relied on ATX (12). To this effect, ATX-dependent transformation of LysoPC into LysoPA promoted the expression of IL6 and BMP2, which, in turn, triggered the mineralization of VIC cultures. In LDLR^−/−^/apoB^100/100^/IGFII mouse, the administration of LysoPA enhanced the expression of BMP2 and promoted a faster progression of aortic valve stenosis (12). Recently, Bouchareb et al. also showed that VIC-derived ATX associates with platelets, which are recruited to the aortic valve during CAVD, and produces LysoPA (53). Hence, both lipoproteins and activated platelets are potential sources of LysoPA in the aortic valve during CAVD.

Nsaibia et al. showed in patients that circulating ATX activity was independently and positively associated with CAVD risk (OR:1.57) (54). Of interest, a significant interaction term was found between ATX activity and Lp(a) level. Stratified analysis showed that an elevated ATX activity and Lp(a) level (≥50 mg/dL) increased the risk of CAVD by 3.5-fold (54). Taken together, these data indicate that ATX is carried by Lp(a) and is also secreted by VICs and contributes to produce LysoPA with pro-inflammatory and osteogenic activities.

## PLPP3: a Negative Regulator of LysoPA

**Figure 2:**
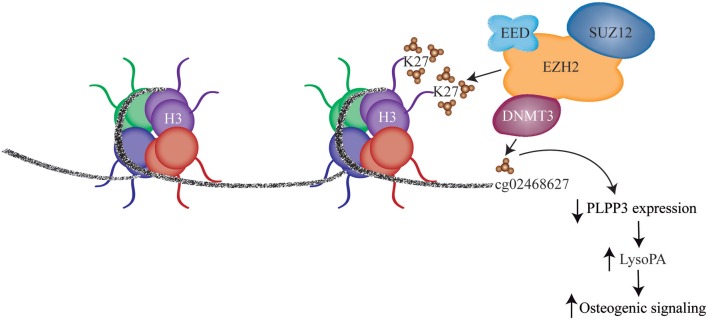
DNA methylation and H3K27 trimethylation in intronic enhancer results in decreased *PLPP3* expression and leads to increased LysoPA level, which promotes osteogenic signaling (With permission from Mkannez et al. (55). EED, embryonic ectoderm development; SUZ12, SUZ12, polycomb repressive complex 2 subunit; EZH2, enhancer of zeste 2 polycomb repressive complex 2 subunit; DNMT3, DNA methyltransferase 3 like.

Phospholipid phosphatases (PLPPs) are membrane-associated enzymes involved in the degradation of LysoPA. As such, PLPPs, which reside at the cell membrane along with LysoPA receptors (LPARs), are key modulator of LysoPA signaling in cells. Transcriptomic analysis of control non-mineralized and mineralized aortic valves showed that among the PLPP enzymes PLPP3 was significantly downregulated during CAVD (55). Analysis in independent series of tissues showed that mRNA encoding for *PLPP3* was decreased by 49% in mineralized AVs. These data were corroborated by measuring PLPP activity, which was also reduced by 31% in mineralized AVs (55). Of note, the level of LysoPA in valves with less expression of *PLPP3* (median value) was increased by 1.5-fold. Mapping provided evidence that a dysregulated epigenetic process was impinging on the expression of *PLPP3* during the mineralization of cusps. DNA methylation mapping showed that CpG methylation in intron 1 (cg02468627) of *PLPP3* was inversely associated with the expression of this gene and was increased in mineralized portion of explanted aortic valves (55). This CpG site is located within a mammalian interspersed repeat (MIR) transposon, a highly conserved repeat element that often harbors enhancers. In this region, the level of monomethylation on lysine 4 of histone 3 (H3K4me1), an histone mark of enhancer, was elevated in aortic valve cusps. Reporter assay confirmed that intron 1 had enhancer activity. Further epigenetic mapping showed an increased level of trimethylation on lysine 27 of histone 3 (H3K27me3) at the intron 1 of *PLPP3* in mineralized aortic valves. H3K27me3 is a histone repressive mark often associated with increased level of CpG methylation. Hence, these findings raised the hypothesis that increased CpG methylation at this site could be a causal event in downregulating the expression of *PLPP3* (Figure 2). Epigenetic editing with clustered regularly interspersed short palindromic repeats (CRISPR)-Cas9 fused with DNA methyltransferase (DNMT) showed that increased CpG methylation at intron 1 of *PLPP3* reduced gene expression by 38% (55). These findings prompted further functional testing to verify if lower expression of *PLPP3* in VICs may promote/exacerbate osteogenic transition induced by LysoPA. Short interfering RNA-mediated knockdown of *PLPP3* in VICs, promoted an accrued response to LysoPA with increased expression of *ALPL* and higher deposition of mineralized matrix. These findings underscored that an epigenetic process downregulates the expression of *PLPP3* during CAVD and is conducive to elevated LysoPA signaling.

## LysoPA and Signaling Cascade in VICs

LysoPA is a bioactive lipid-derived metabolite with a vast array of biological activities. At least 6 different LPARs mediate the action of LysoPA. Fine-grained molecular analysis recently deciphered the signaling cascade whereby LysoPA and OxPLs promoted inflammation and osteogenesis in VICs (56). We highlighted that LPAR1 was highly expressed in human mineralized valves and promoted the expression of *COL1A1* and the mineralization of VIC cultures through a nuclear factor kappa B (NFκB) pathway. In VICs, LPAR1 activated RhoA and Rho kinase (ROCK), which promoted a robust expression of NFκB-dependent genes such as IL6 and IL8. In addition, we found that *BMP2*, a morphogenetic protein that drives osteogenesis, was overexpressed downstream of LPAR1-NFκB. Analysis showed that promoter region of *BMP2* contained κB responsive elements and that LysoPA promoted the phosphorylation of p65 on serine 536 (p65 S536) (56). Of interest, phosphorylated p65 S536 was recruited to the promoter of *BMP2* in chromatin immunoprecipitation (ChIP) assay. During NFκB signaling the inhibitor of κB (IκB) is rapidly induced and transits to the nucleus where it competes with p65 for κB sites. This process ensures a negative feedback loop to control the inflammatory response mediated by the NFκB pathway. However, phosphorylation of p65 S536 is not sensitive to the nuclear inhibition provided by IκB. Hence, genes dependent on phosphorylated p65 S536 are less sensitive to negative retro-feedback and experienced sustained expression. Experiments carried out with constitutively active mutant IκB super repressor (IκBα SS32-36AA) showed that LysoPA-induced expression of *BMP2* was not sensitive to the repression (56). These data thus indicate that LysoPA-induced activation of *BMP2* is dependent on phosphorylated p65 S536 and is not responsive to the negative feedback regulation provided by IκB. In a mouse model, pharmacological inhibition of LPAR1with Ki16425 reduced the progression of aortic stenosis and downregulated the expression of BMP2 in aortic valve cusps (56). In this model, the administration of Ki16425 was not associated with significant modification in the lipid profile. It thus suggests that the protection provided by the pharmacological inhibition of LPAR1 was likely mediated by direct valvular effect and decreased osteogenic signaling in the aortic valve.

## Novel Therapeutic Opportunities in CAVD

Randomized control trials (RCTs) conducted with statins have been negative in CAVD and have questioned the role of lipoproteins as causal factors in CAVD. However, the strong genetic signal for *LPA* in CAVD, which has been validated in different cohorts, has provided considerable interest to understand the role of lipoproteins in CAVD. MR studies showed that Lp(a) is causally associated with CAVD. Thus, these data strongly suggest that interventions aimed to modify the level of Lp(a) could provide therapeutic benefit (57). The failure of statins in RCTs is probably multifactorial (58). For instance, pleiotropic properties of statins include a pro-osteogenic effect and also this class of LDL-lowering drugs increases the level of Lp(a) significantly (25). Recently, oligoantisense therapy that lowers Lp(a) level by more than 80% have been developed and could be tested in RCT to reduce the progression of aortic stenosis (59). Also, inhibitors of proprotein convertase subtilisin/kexin type 9 (PCSK9), which provides drastic reduction of LDL and a moderate reduction of Lp(a) (20–25%), could be tested in CAVD (60). In addition, promising data showed a reduction of aortic valve mineralization in *Ldlr*^−/−^ mice expressing a natural antibody against OxPL (EO6) (61). Further exploration of blocking antibodies directed at OxPL epitopes in humans could lead to novel therapies. On the other hand, considering the identification of enzymatic pathways that mediate OxPL-induced mineralization of the aortic valve, several promising drugs in development are of interest and should be valuated in pre-clinical models as well as in RCTs. In this regard, inhibitors of ATX and LPARs are in different phases of development and could be evaluated as potential therapeutic agents in CAVD (62).

## Conclusion

Genetic studies have underscored that Lp(a) is causally associated with CAVD. Molecular analyses have provided evidence that interaction between ATX and Lp(a) is promoting inflammation and osteogenic reprograming in VICs. Dysregulation of LysoPA signaling in mineralized aortic valve includes a higher and a lower expression of *LPAR1* and *PLPP3* respectively. LPAR1 promotes a signaling cascade that culminate in the phosphorylation of p65 S536 and a sustained expression of *BMP2*, a key driver of osteogenic transformation in VICs. On the other hand, epigenetically reprogrammed VICs at a transposon-based enhancer is a proximal event that contributes to lower *PLPP3* and to promote/exacerbate LysoPA signaling in VICs. Together, these data suggest that lipoprotein metabolism during CAVD is perturbed and promotes inflammation and mineralization. Further work and elucidation of key processes in lipoprotein metabolism during CAVD could lead to the development of novel therapies.

## Data Availability

The datasets for this manuscript are not publicly available. Requests to access the datasets should be directed to Patrick Mathieu.

## Author Contributions

All authors listed have made a substantial, direct and intellectual contribution to the work, and approved it for publication.

### Conflict of Interest Statement

The authors declare that the research was conducted in the absence of any commercial or financial relationships that could be construed as a potential conflict of interest.
